# Prognosis and clinical features analysis of EMT-related signature and tumor Immune microenvironment in glioma

**DOI:** 10.5937/jomb0-39234

**Published:** 2023-01-20

**Authors:** Zheng Xiao, Xiaoyan Liu, Yixiang Mo, Weibo Chen, Shizhong Zhang, Yingwei Yu, Huiwen Weng

**Affiliations:** 1 Southern Medical University, Zhujiang Hospital, Department of Neurosurgery, Guangzhou, China; 2 Jinan University, The First Affiliated Hospital of Jinan University, Department of Neurology, Guangzhou, China; 3 Jinan University, The Fifth Affiliated Hospital of Jinan University. Department of Neurology, Heyuan, China; 4 The Second Affiliated Hospital of Guangzhou University of Chinese Medicine, Guangzhou, China; 5 The First Affiliated Hospital of Sun Yat-sen University, Department of Oncology, Guangzhou, China

**Keywords:** glioma, epithelial mesenchymal transition, tumor microenvironment, immunotherapy, risk score, gliom, epitelna mezenhimalna tranzicija, tumorsko mikrookruženje, imunoterapija, skor rizik

## Abstract

**Background:**

As the most common primary malignant intracranial tumor, glioblastoma has a poor prognosis with limited treatment options. It has a high propensity for recurrence, invasion, and poor immune prognosis due to the complex tumor microenvironment.

**Methods:**

Six groups of samples from four datasets were included in this study. We used consensus ClusterPlus to establish two subgroups by the EMT-related gene. The difference in clinicopathological features, genomic characteristics, immune infiltration, treatment response and prognoses were evaluated by multiple algorithms. By using LASSO regression, multi-factor Cox analysis, stepAIC method, a prognostic risk model was constructed based on the final screened genes.

**Results:**

The consensusClusterPlus analyses revealed two subtypes of glioblastoma (C1 and C2), which were characterized by different EMT-related gene expression patterns. C2 subtype with the worse prognosis had the more malignant clinical and pathology manifestations, higher Immune infiltration and tumor-associated molecular pathways scores, and poorer response to treatment. Additionally, our EMT-related genes risk prediction model can provide valuable support for clinical evaluations of glioma.

**Conclusions:**

The assessment system and prediction model displayed good performance in independent prognostic risk assessment and individual patient treatment response prediction. This can help with clinical treatment decisions and the development of effective treatments.

## Abstract (sr)

**Uvod:**

Kao najčešći primarni maligni intrakranijalni tumor, glioblastom ima lošu prognozu sa ograničenim mogućnostima lečenja. Ima visoku sklonost ka recidivu, invaziji i lošu imunološku prognozu zbog kompleksnog mikrookruženja tumora.

**Metode:**

U ovu studiju uključeno je šest grupa uzoraka iz četiri skupa podataka. Koristili smo konsenzus ClusterPlus da uspostavimo dve podgrupe pomoću gena povezanog sa EMT Razlika u kliničko-patološkim karakteristikama, genomskim karakteristikama, imunološkoj infiltraciji, odgovoru na lečenje i prognozama je procenjena pomoću više algoritama. Korišćenjem LASSO regresije, multifaktorske Cok analize, stepAIC metode, konstruisan je model prognostičkog rizika na osnovu finalnih skrining gena.

## Introduction

The glioblastoma (GBM) accounts for nearly half of all malignant brain tumors [1] [2] [3]. The median survival time of patients with glioblastoma is only 12-14 months after a comprehensive treatment [1] [2] [3] [4] [5]. Because tumor cells differ greatly in terms of genetics and epigenetics, treatment is challenging. At present, strategies, such as surgical resection, postoperative radiotherapy, and temozolomide chemotherapy, for the treatment of glioma cannot fundamentally improve the prognosis of patients with glioma [1] [2] [4]. The interconnectedness of tumor cells and other cells in the microenvironment plays a significant role in promoting the development of glioblastoma. Therefore, further exploration of the nosogenesis of glioblastoma and the development of innovative methods are urgently needed [6] [7] [8] [9].

In recent years, significant progress has been made in the field of cancer immunotherapy, and the FDA has already licensed more than 50 indications of chimeric antigen receptor transduced T cells, bi-specific T cell binding antibodies, and immune checkpoint blocking antibodies for cancer treatment [10] [11]. Although immunotherapy has shown great effectiveness in various cancers, it has not achieved the desired effect in treating gliomas. Multiple phase III clinical studies of gliomas have failed [12] [13]. In recent studies, it has been found that cells undergoing EMT can also regulate antitumour immunity. Cells from the adaptive immune system exist in tumor-related matrix, which can eliminate tumor cells, while cells from mesenchymal cells resist this process. Consequently, EMT-induced quasi-mesenchymal state has significant implications for the field of clinical oncology, as both chemotherapy and immunotherapy are more difficult to treat when they exist [3] [6] [8].

In this study, we analyzed the different expression patterns of the related genes in the glioblastoma EMT pathway through bioinformatic methods. Subtypes with different survival outcomes, functional characteristics, and clinical features were also identified. Differences in the abundance of immune cell infiltrates, genes associated with immune checkpoints, and cell stemness index among different subtype patients were analyzed to determine the effectiveness of immunotherapy. Finally, we screened out the prognosis-related genes involved in EMT pathway in patients with glioblastoma and established a risk model with good predictive efficacy and prospects with clinical application. Our study aims to provide a reference for the progress of precision therapy and immunotherapy for patients from the perspective of exploring tumor microenvironment heterogeneity in glioblastoma.

## Materials and methods

### Data sources and pre-processing

RNAseq or microarray expression profiles were collected from TCGA (https://tcga-data.nci.nih.gov/tcga/), CGGA (CGGA693 and CGGA325, http://www.cgga.org.cn/), GEO (GSE4271 and GSE7696, https://www.ncbi.nlm.nih.gov/geo/), and Rembrandt (http://caintegrator-info.nci.nih.gov/rembrandt). Clinical follow-up information related to the above four datasets was also downloaded. The ComBat function of the sva package was used to remove the batch effects among four data sets, that is, TCGA, GSE4271, GSE7696 and Rembrandt [14]. Then they were combined into one data set, hereinafter referred to as Array. The same method was used to remove the batch effects among the two RNA-Seq date sets of CGGA693 and CGGA325. Then they were combined into one data set, hereinafter referred to as RNASeq. The relevant clinical information of the sample was listed in Table 1.

**Table 1:** The relevant clinical information of the sample.

Feature	TCGA	GSE4271	GSE7696	Rembrandt	CGGA693	CGGA325
Event						
Alive	77	62	15	9	40	13
Dead	447	15	65	137	197	124
Gender						
Female	205	25	21	48	98	50
Male	319	52	59	74	139	87
NA				24		
Age						
>55	312	14	28	71	88	37
<=55	212	63	52	71	149	100
Unknown				4		
Chemotherapy						
NO	136					
YES	388					
NA						
Radiotherapy						
NO	2				32	32
YES	3				193	100
NX	519				12	5
TMZ						
NO					27	34
YES					199	99
NA					11	4
IDH.status						
Mutant	34				45	39
WT	375				182	98
NA	115				10	
X1p.19q						
Codel	2			3	12	7
non-codel	502			65	197	127
NA	20				28	3
KPS						
20	2					
40	13					
60	80					
70	4					
80	231					
90	5					
100	57					
NA	132					
MGMT						
Methylated	157		44		104	65
Unmethylated	191		34		89	70
NA	176		2		44	2
Original. Subtype						
Classical	143					
G-CIMP	39					
Mesenchymal	155					
Neural	83					
Proneural	99					
NA	5					
Transcriptome. Subtype						
CL	155					
ME	173					
NE	61					
PN	107					
NA	28					

### Consistency clustering analysis

We used the R package ConsensusClusterPlus (V 1.52.0) to select the optimal typing and type the RNASeq cohort [15]. We then analyzed whether the survival curves (KM curves) differed between molecular subtypes. The t-distributed stochastic neighbor embedding (t-SNE) method was used to verify the subtype assignment using mRNA expression data from the EMT gene described above. The same approach was used to validate the isoform assignment for the Array cohort. In addition, differences in the distribution of clinical features between the molecular subtypes were compared. A chi-square test was performed and P < 0.05 was considered significant.

### Sample Gene Set Enrichment Analysis (ssGSEA)

Pathway gene features in h.all.v7.4.symbols.gmt were assessed using the R package GSVA and GSEABase (V1.50.1). Pathway scores were then compared between subtypes using the rank-sum test. In addition, based on previous studies, immune checkpoints were selected for comparison between subtypes.

### Characterization of glioma subtypes

Differentially expressed genes (DEGs) between glioma subtypes were analyzed and identified using the R software package limma [16]. Genes with |log2FC|> 1 and false discovery rate (FDR) < 0.05, were defined as DEGs. R software package cluster Profiler was used to perform Gene Ontology (GO) functional annotation and KEGG pathway enrichment analysis for differentially upregulated and differentially downregulated genes, respectively, filtered by a threshold of FDR < 0.05.

### Analysis of GSEA between molecular subtypes

We used the GSEA function in clusterProfiler to verify the function and plausibility of the subtypes by using the enriched pathways of the h.all.v7.4.symbols.gmt and c2.cp.kegg.v7.4.symbols.gmt pathway gene set analysis subtype.

### Immunological microenvironmental analysis of molecular subtypes

We used five methods, including MCP-counter [17], TIMER [18], ESTIMATE [19], ssGSEA [20], and CIBERSORT [21] to assess immune infiltration in the RNASeq and Array cohorts, and subsequently compared the differences in immune cell scores between different subtypes. The Kruskal-Wallis test was performed for a difference analysis between subtypes. Twenty-eight immune cell markers included in the ssGSEA algorithm were obtained from a previous study.

### Analysis of stem cell indices between subtypes

The stemness indices were calculated from previous studies, where mRNAsi is an index calculated based on expression profile data and ranges from 0 to 1, with values closer to 1 indicating less differentiated cells and stronger stem cell characteristics. Differential analysis of mRNAsi between different isoforms was performed using the Kruskal-Wallis test [22] [23] [24] [25].

### Predicting the efficacy of immunotherapy and targeted therapies for each subtype

We used the TIDE algorithm to predict the efficacy of subtype immune checkpoint blockade therapy [26] [27]. The Gene Pattern category mapping (SubMap) was used to compare the similarity of gene expression profiles between the available data of immunotherapy patients and the two subtypes to indirectly predict the efficacy of subtype immunotherapy [28] [29]. In addition, we used the R package pRRophetic to predict the sensitivity of the IC50 of the drugs cisplatin, paclitaxel, sorafenib, erlotinib, crizotinib and temozolomide in our molecular subtypes.

### Construction and validation of prognostic models

Using RNASeq cohort as the training data set, prognosis-related genes in the RNASeq and Array cohort were used as targets for the study. The filtered genes were further compressed using the least absolute shrinkage and selection operator (LASSO) regression to reduce the number of genes in the risk model. The remaining genes were subjected to multifactor Cox analysis and the number was further reduced using the stepAIC method. The final screened genes were prognosis-associated, and the prognostic risk model was calculated as follows: 


(1) }{}RiskScore = \sum\limits _{i=1}^{n} coef(i)^* \ gene(i)

where, coef (i) represents the coefficient of the i^th^ gene, and gene (i) represents the expression of the i^th^ gene. A RiskScore value was calculated for each sample, using the median as the split point. The samples were then divided into two groups of high and low risk. The Array cohort and TCGA dataset were used as test sets, and the prognostic risk model prediction performance was validated in the same way. We also compared the differences in RiskScore between the different clinical characteristic groupings.

## Results

### Two different vascular gene expression patterns in glioma

**Figure 1:**
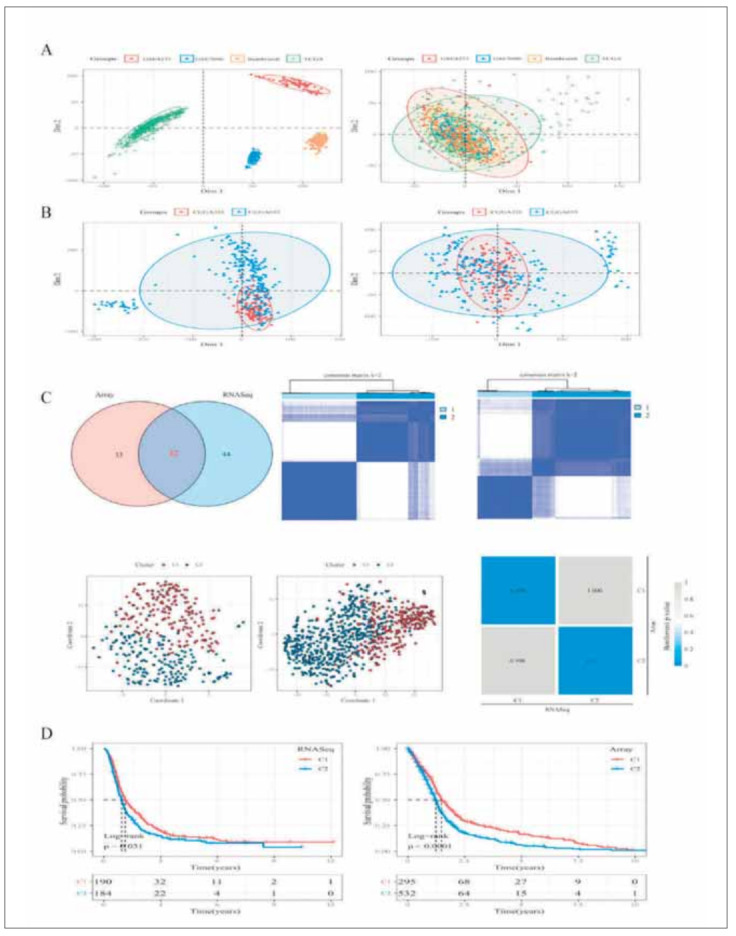
Expression of CTSA in glioma. (A) CTSA expression in different types of cancer was detected with TIMER database. (B) Increased or decreased of CTSA in glioma cancer compared to normal tissues in the GEPIA database. (C) Expression level of CTSA in glioma cancer was detected with UALCAN database. (D) Expression level of CTSA in glioma cancer tissues and normal tissues were determined with TCGA database.

Two hundred genes related to EMT were selected from existing studies. The survival data were used to conduct univariate Cox analysis. We selected P<0.05 as the threshold value for filtering. There are 76 genes related to the prognosis in RNASeq. There are 65 genes related to the prognosis in Array data. The intersection of them was selected for cluster analysis and 32 genes were obtained in total (Figure 1A and Figure 1B).

Molecular typing was performed on the RNASeq dataset using the R package Consensus-ClusterPlus. The consensus matrix heat map maintained clear and sharp boundaries (Figure 1C) when k = 2, indicating that the clustering of the samples was stable, robust, and consistent with the two-dimensional t-SNE distribution pattern (Figure 1C). Subsequently, the datasets of Array samples were treated using the same method for cluster analysis, and the same conclusion as the RNASeq dataset was obtained (Figure 1C). It was shown that two subclasses of glioma molecules with different EMT gene expression patterns existed and that subtype C1 had the better prognosis and C2 had the worse (Figure 1D).

### Clinical features of different subtypes

**Figure 2:**
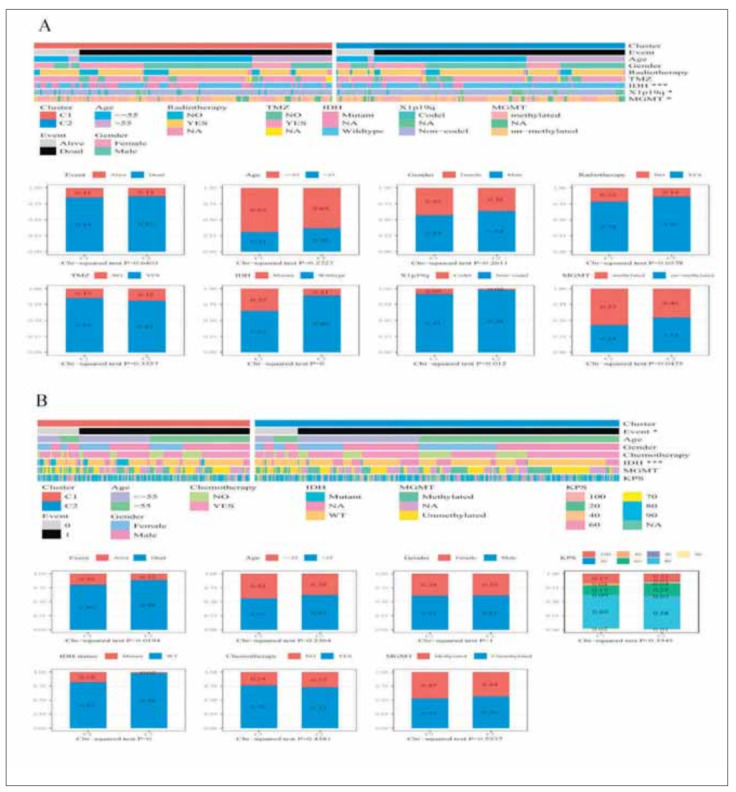
Comparison of clinical information between subtypes. (A) Upper panel: Comparisons of clinical-pathological characteristics among angiogenesis subtypes in the RNASeq cohort. Lower panel: Comparisons of event, age, gender, IDH status, grade, 1p/19q codeletion status, and MGMT promoter status among angiogenesis subtypes in the RNASeq cohort. (B) Upper panel: Comparisons of clinical-pathological characteristics among angiogenesis subtypes in the TCGA cohort. Lower panel: Comparisons of event, age, gender, KPS, IDH status, and MGMT promoter status among angiogenesis subtypes in the TCGA cohort.

We compared the clinical differences among different molecular subtypes in RNASeq (Figure 2A). The results were as follows: 1) The Dead rate in the C2 subtype with the worse prognosis was higher; 2) The proportions of Age, Gender, IDH, X1p19q, and MGMT were different in different subtypes. The proportion of wild-type IDH1 in C1 subtype with a better prognosis was lower than that in C2, and the results were in line with our expectations. It is suggested that IDH1 mutant and wild-type tumor cells may exhibit different phenotypes, during epithelial interstitial processes. In addition, X1p19q co-deletion expression in C1 subtype is also higher, consistent with better oligodendrocyte prognoses. It suggests that oligodendrocytes show greater affinity for the C1 subtype during EMT. In addition, the TCGA data set was characterized by abundant clinical information and we compared the distribution differences of the clinical features among different subtypes in the TCGA data set (Figure 2B).

### Analysis of differentially expressed genes

**Figure 3:**
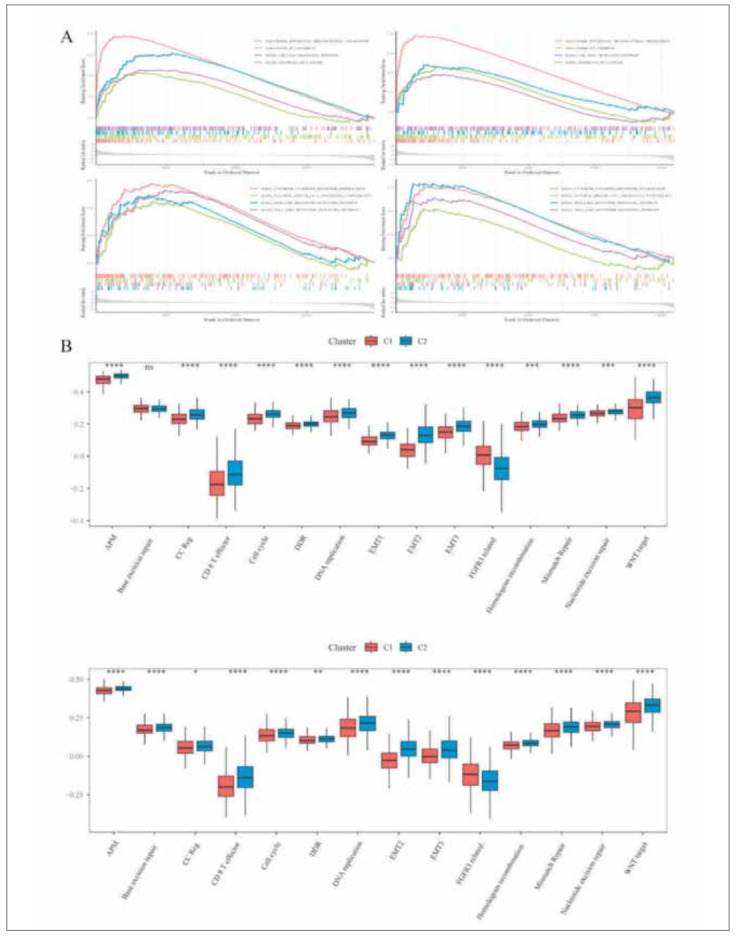
Pathway analysis of differentially expressed genes among different subtypes. (A) Differentially enriched pathways analyses by GSEA of subtype C1 or C2 in the RNASeq and Array cohorts. (B) An ssGSEA algorithm was applied to quantify the human signatures between Subtype C1 and C2 in the RNASeq and Array cohorts (^*^represents P < 0.05, ^**^represents P < 0.01, ^***^represents P < 0.001, ^****^represents P < 0.0001).

We used the limma package to calculate the differential genes of Array and RNASeq, respectively. We took FDR < 0.05 and |FC| > 1.5 as the threshold value to identify differential genes. We conducted KEGG path analysis and GO functional enrichment analysis on the differential genes among subtypes. The results showed that Focal adhesion, TNF signaling pathway, ECM−receptor interaction in the C2 subtype adhered to cells and migrated and the path expression of tumor invasion had a rise. The GSEA algorithm also confirmed these results (Figure 3A). In order to further study the characteristics of the subclass, we used ssGSEA in the GSVA algorithm to select and quantify 15 signs related to human signatures [30] [31]. We found the 15 signs also showed significant differences among subtypes. The score of the C2 subtype was significantly higher than that of C1. The results were consistent in RNASeq and Array (Figure 3B).

### Analysis of immune cell infiltration and treatment response

**Figure 4:**
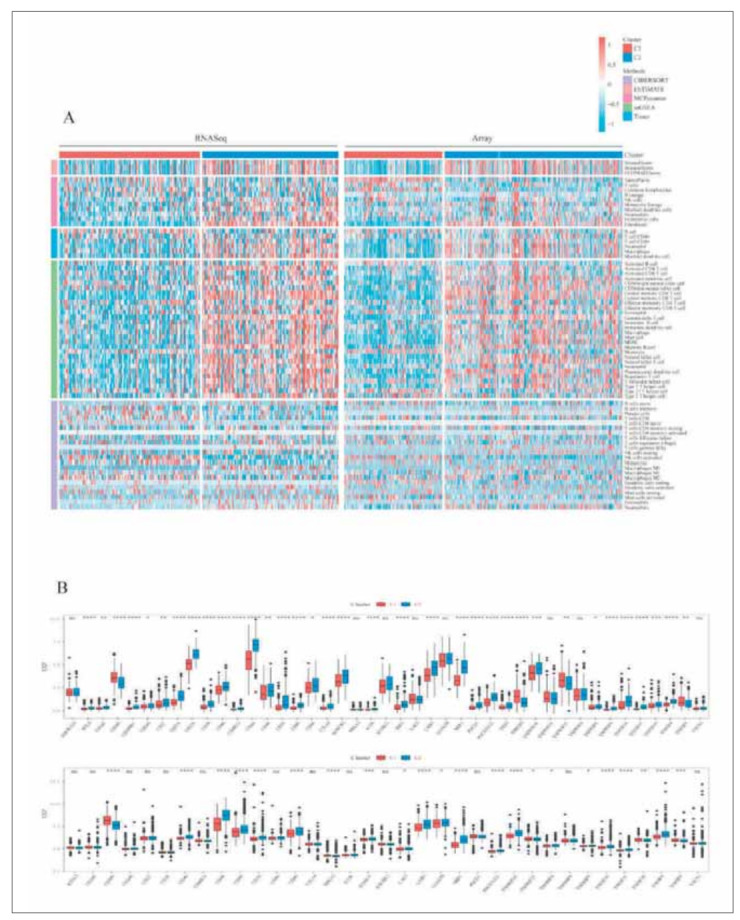
Immune score and immune checkpoints. (A) The heat map of immune cell infiltration scores evaluated by four immune evaluation software (MCP-counter, ESTIMATE, ssGSEA, EPIC) on angiogenesis subtypes in the RNASeq and Array cohorts. Blue represents low enrichment scores, and orange represents high enrichment scores. (^*^represents P < 0.05, ^**^represents P < 0.01, ^***^represents P < 0.001, ^****^represents P <0.0001). (B) Differential expression of immune checkpoint molecules between Subtype C1 and C2 in the RNASeq (Upper panel) and Array cohorts (Lower panel).

**Figure 5:**
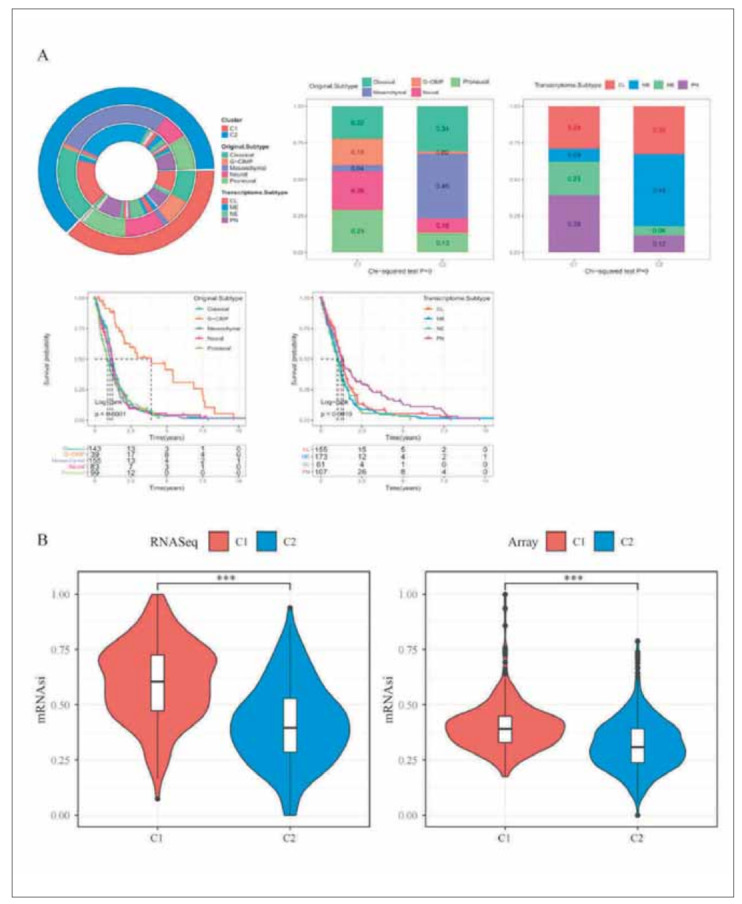
Difference in glioma immune subtype distribution and stemness score. (A) Upper left panel: Circle graph comparison among subtypes (The outer layer is our molecular subtype, the second layer is the existing Original Subtype, the third layer is the existing Transcriptome Subtype, and the fourth layer is the existing Pan Glioma RNA Expression Cluster). Upper right panel: The distribution comparison of existing Original Subtype, Transcriptome Subtype, Pan Glioma RNA Expression Cluster in our molecular subtype distribution comparison. Lower panel: Survival curve of subtypes of existing Original Subtype, Transcriptome Subtype, Pan Glioma RNA Expression Cluster. (B) Comparisons of mRNAsi in EMT subtypes in the RNASeq and Array cohorts.

**Figure 6:**
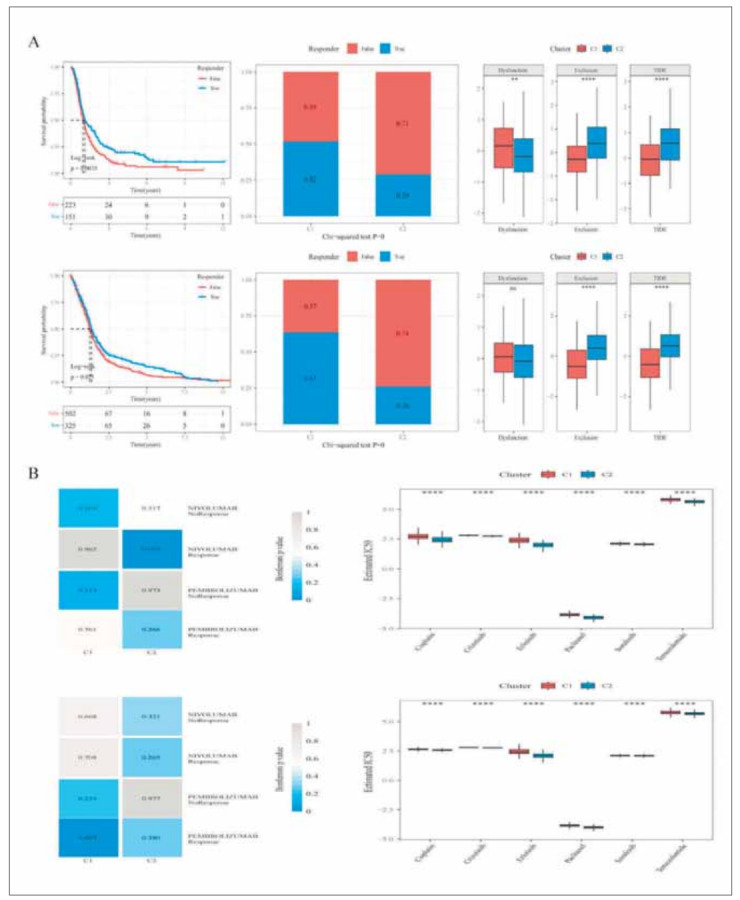
TIDE analysis for treatment response and the sensitivity of different subtypes to chemotherapeutic drugs in the RNASeq and Array cohorts. (A) left panel: K-M survival analysis of no responders and responders subtypes. Middle panel: Comparisons of the proportions of no responders and responders to immunotherapy among C1 and C2 subtypes. Right panel: Comparisons of TIDE, Dysfunction, and Exclusion score among EMT subtypes. (B) left panel: SubMap analysis for predicting the immunotherapy data among different EMT subtypes. Right panel: The comparison of IC50 of different drugs among EMT subtypes.

To further analyze the differences in immune microenvironment between subtypes, we used five methods namely, MCP-counter, TIMER, ESTIMATE, ssGSEA, and CIBERSORT to assess immune infiltration in the RNASeq and Array cohorts (Figure 4A) and found that the C2 subtype with the worse prognosis had the higher immune score, while the C1 subtype with the better prognosis had the lower immune score. The evaluation results of the different softwares are consistent across datasets and with those of our previous study. We found the genes related to immune checkpoints from previous studies and compared these genes' expressions in different subtypes. The results were as follows: 1) In RNASeq, 35 (81.4%) genes at 43 immune checkpoints had significant different expressions in subtypes. 2) In Array, 22 (62.9%) genes at 35 immune checkpoints had significant different expressions in subtypes. This suggested that there may be differences in immunotherapy between subtypes. In addition, we found that the expressions of most immune checkpoints were higher in the C2 subtype, including CTLA4, PDCD1, IDO1, and CD40 (Figure 4B). Then we analyzed the sample distribution between C1, C2 subtypes and the existing immune molecular subtypes (Original. Subtype and Transcriptome. Subtype) [32]. Both the G-CIMP subtype in Original. Subtype and the PN subtype in Transcriptome. Subtype accounted for much higher proportions in the C1 subtype than in the C2 subtype (Figure 5A). We calculated mRNAsi based on the data of the expression profile and compared them among subtypes. The results showed that the mRNAsi of the C2 subtype with the worse prognosis was significantly lower than that of the C1 subtype with the better prognosis in both RNASeq and Array (Figure 5B). The TIDE algorithm was used to predict the responsiveness of immunotherapy in glioma patients in the two datasets by comparing the proportion of treatment response and the TIDE scores in different subtypes. As shown in the Figure 6A: 1) In RNASeq, the prognosis of the group which predicted the response to treatment is better. The TIDE score and Exclusion score of the C2 subtype were higher than those of the C1 subtype. The Dysfunction score of the C1 subtype was higher than that of the C2 subtype. There were significant differences in the Responder results among subtypes. 2) The similar results were obtained from Array. We compared the expression profiles of the two GBM subtypes (C1 and C2) with another published data set of GSE93157. This data set included the patients that were treated with NIVOLUMAB and PEMBROLIZUMAB. The C2 subtype in RNASeq was significantly correlated with the expression profile of the NIVOLUMAB response group, suggesting that patients in the C2 group had a more promising response to NIVOLUMAB treatment. At the same time, the C1 group was significantly correlated with the expression profile of the PEMBROLIZUMAB response group in the subtypes of Array, suggesting that patients in the C1 group of Array had a more promising response to PEM-BROLIZUMAB treatment (Figure 6B). In addition, we calculated the IC50 of Cisplatin, Paclitaxel, Sorafenib, Erlotinib, Crizotinib, and Temozolomide. We compared their differences among different subtypes and found that the six drugs all showed significant differences among the subtypes in RNASeq and Array. What's more, they showed the consistent trend among different subtypes. The C2 subtype was more sensitive to these drugs (Figure 6B).

### Model construction and application

**Figure 7:**
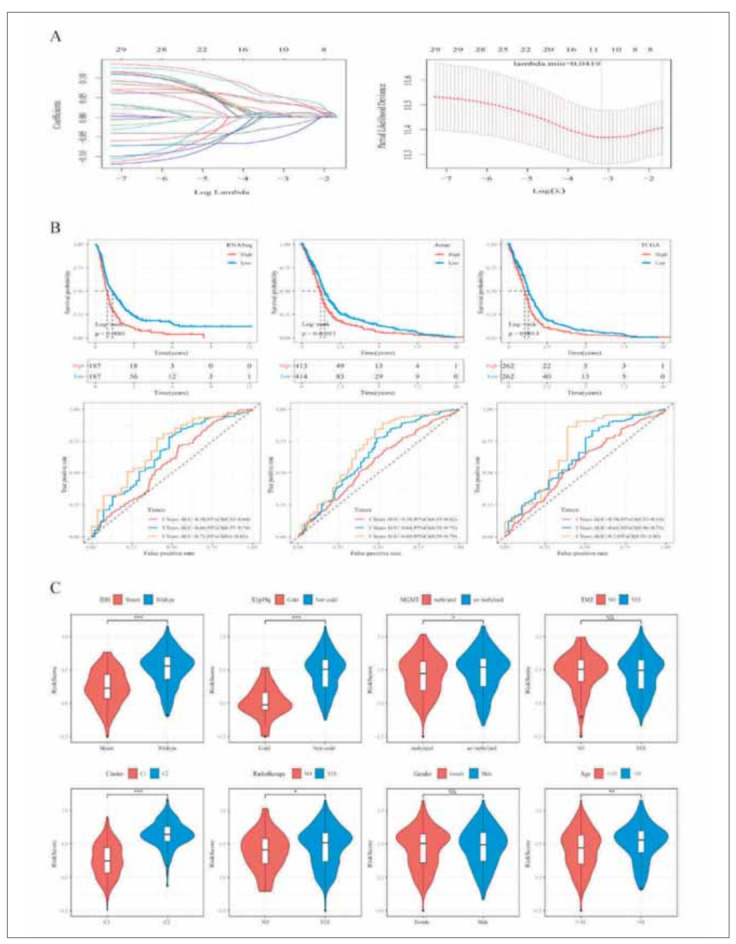
Risk score calculation to predict prognosis (A) LASSO regression analysis. (B) Upper panel: Survival curve of high and low RiskScore in the RNASeq, Array, and TCGA cohorts. Lower panel: ROC curves of the RiskScore in the RNASeq, Array, and TCGA cohorts. (C) Comparisons of RiskScore between IDH status, 1p/19q codeletion status, MGMT promoter status, TMZ, Cluster, Radiotherapy, gender, and age.

The glmnet package of R software was adopted to conduct LASSO logistic regression analysis on RNASeq. The 10-fold cross-validation was adopted to build a model (Figure 7A). The confidence interval of each lambda was analyzed as shown. When lambda=0.0419, the model was optimal, so 10 genes in this case were selected for further analysis. Multivariate Cox analysis was conducted on the 10 genes. At the same time, the stepAIC method was used to reduce the number of genes. Finally, four genes were used in our model. Based on the calculation formula of RiskScore, the risk score of each sample in the RNASeq training data set was obtained. The samples were divided into two groups based on the median, that is, the high-risk group and the low-risk group. There were significant differences in the survival curves between the two groups (Figure 7B). After Array data and TCGA data were validated, it was found that there were also significant differences in survival curves between the high-risk group and the low-risk group. In addition, the 1-year, 3-year, and 5-year ROC curves of RiskScore in the data set were calculated and the AUC values were also good (Figure 7B).

Further analysis revealed that in the RNASeq dataset, RiskScore was not significantly different between the sex subgroups, but was significantly different between the IDH, X1p19q, MGMT, grade, age, and molecular subtypes (P < 0.05) (Figure 7C). RiskScore was significantly higher in the worse prognosis C2 subtype than in the better prognosis C1 subtype (Figure 7C).

**Figure 8:**
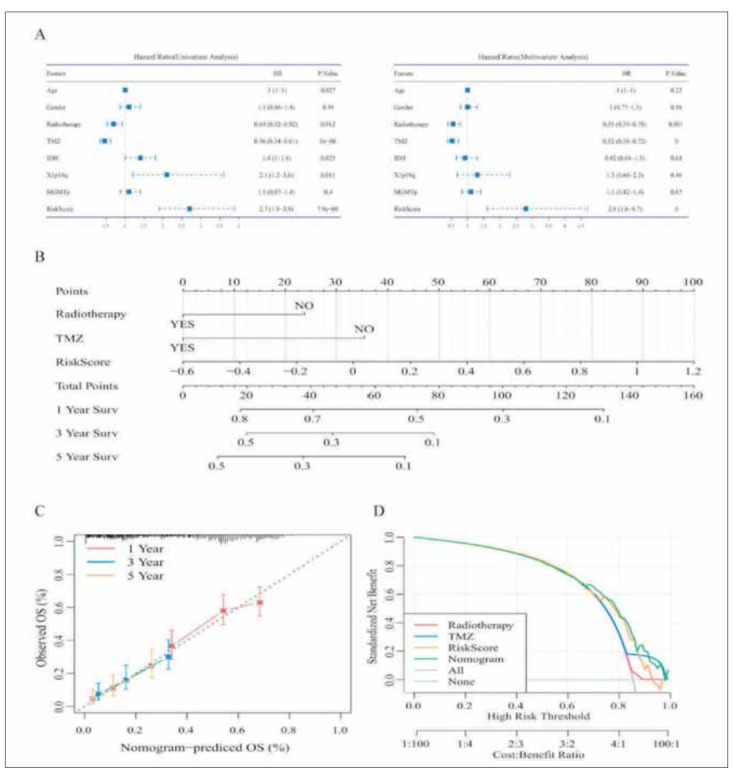
Clinical applications of the riskScore model. (A) Univariate and Multivariate (B) Nomogram. (C) Correction nomogram. (D) Clinical characteristics and DCA of RiskScore.

To identify the independence of the RiskScore model in clinical applications, we analyzed the associated HR, 95% CI of HR, and P-value in the RNASeq dataset using univariate and multifactorial Cox. We systematically analyzed the patient's age, sex, IDH, X1p19q, MGMT, and RiskScore, and found that our RiskScore was an independent prognostic factor (Table 2). Column line plots were then used to present the risk model results visually and efficiently. The independent prognostic factors in the RNASeq dataset were used to construct the column line graph model (Figure 8A), and the results showed that the RiskScore feature had the greatest impact on survival prediction (Figure 8B). This indicates that the fourgene-based risk model can predict prognosis better. The column line graphs (1-, 3-, and 5-year) were corrected and presented in Figure 8C. The prediction model was evaluated using DCA (Figure 8D). It was demonstrated that RiskScore has good performance, while weighted clinical features (nomogram) have better results.

**Table 2:** The Risk factor and multi-factor Cox regression analysis.

Gene	Coef	HR	HR<br>(lower, 0.95)	HR<br>(upper, 0.95)	P
SPP1	0.063	1.066	1.005	1.130	0.034
SPOCK1	-0.112	0.894	0.826	0.968	0.005
PLOD2	0.099	1.104	0.992	1.229	0.070
CRLF1	-0.052	0.949	0.884	1.019	0.147

## Discussion

The exogenous components and intrinsic mechanisms of tumor cells determine the immunosuppressive state of the glioma microenvironment, which greatly limits the efficiency of immunotherapy. The data show that EMT is common before, during, and after treatment. When EMT is caused by treatment, it often leads to treatment resistance or recurrences of cancer. The coexistence of EMT and immune response has been shown to be emerging evidence in recent years. Research on the interaction between EMT and glioma immune escape, as well as their impact on glioma tumor behavior, is lacking [1] [2] [3] [6] [33]. Our study analyzed the relationships between EMT-related genes and efficiency of immunotherapy and chemotherapy and the prognosis in depth. The study verified our assumption by using patient samples and clinical data from six public datasets, proposing a method to distinguish glioma subtypes based on EMT-related genes. We found two different EMT gene expression patterns in glioma, in which significant differences in molecular marker expression, tissue grade ratio, immune infiltration, and treatment response can be observed in prognosis, indicating that we can identify glioma-related biological characteristics of patients before treatment, then stratify and predict treatment outcomes through the clustering of EMT-related genes.

Considering the previous failure of the treatment of patients with GBM through single drug immunotherapy, an increasing number of combined therapy strategies have been introduced into immunotherapy research for glioma [1] [2] [33] [34]. There are studies indicating that EMT-related molecules trigger the release of immune-regulatory cytokines and chemokines by inducing autophagy in target cells [6] [30]. Furthermore, activating the EMT program alters the formation of an immunological synapse between tumor cells and T cells, thereby dampening T cell priming. EMT not only reduces MHC class I levels on carcinoma cells surfaces, but induces PD-L1 expression which exhausts T cells when it interacts with its cognate receptor [35] [36].

Our study also found different EMT-related genes expression patterns in the therapeutic response to PD-L1, and significant differences in the degree of immune cell infiltration were observed between C1 and C2 subtype. In the C2 subtype with the highest EMT score, a high abundance of CD4+ and CD8+ T cell subsets, NK cells, monocytes, macrophages, and neutrophils was observed. This indicates that considering the subtype classification methods of EMT-related genes as an assessment reference for patient grouping criteria and therapeutic evaluation can improve the efficiency of drugs acting on Wnt or TGF pathway combined with immunotherapy.

Moreover, we established a clinically-assisted assessment system and risk prediction model based on the molecular subtypes of EMT-related genes, which is a useful tool for predicting independent prognostic risk and treatment response of individual patients, providing help for clinical treatment decision-making and developing effective treatment methods. Among the four genes used for modeling, The PLOD2 is an enzyme located in the rough endoplasmic reticulum of the cytoplasm that modifies collagen post-translationally. Proteins of the rest three genes are all Extracellular protein or located on cell membrane. The Spp1 and SPOCK1 are glycoprotein that constructs extracellular matrix. The CRLF1 mRNA is primarily expressed in fibroblasts and can be increased by an inflammatory response. They all show good correlation with prognosis, indicating that by changing the way of EMT, extracellular matrix remodeling and inflammatory reactions in tumors may affect the prognosis of patients. PLOD2, Spp1, SPOCK1 and CRLF1 can also serve as new targets for anti-tumor microenvironment therapy of glioma.

However, this study has some limitations. To verify our results, we used samples from a public database as the validation set to support the conclusions of this study. In the future, we need to expand our experiments to patients with complete clinical and pathological data for clinical verification.

In conclusion, this study demonstrated a subtype classification method based on glioma EMTrelated genes, which can provide a new reference and assessment method for fundamental mechanism research and clinical treatment decision-making in patients with glioma. This improves the precision of tumor treatment with good potential for clinical applications.

## Dodatak

### Conflict of interest statement

All the authors declare that they have no conflict of interest in this work.
